# COVID-19 health policy evaluation: integrating health and economic perspectives with a data envelopment analysis approach

**DOI:** 10.1007/s10198-021-01425-7

**Published:** 2022-01-11

**Authors:** Matthias Klumpp, Dominic Loske, Silvio Bicciato

**Affiliations:** 1 Chair of Production and Logistics Management, Department for Business Administration, Georg-August-University of Göttingen, Platz der Göttinger Sieben 3, 37073 Göttingen, Germany; 2 FOM University of Applied Sciences Essen, Leimkugelstr. 6, 45141 Essen, Germany; 3 Fraunhofer Institute for Material Flow and Logistics IML Dortmund, J.-v.-Fraunhofer-Str. 2-4, 44227 Dortmund, Germany; 4 Interdepartmental Center for Stem Cells and Regenerative Medicine (CIDSTEM), Department of Life Sciences, University of Modena and Reggio Emilia, Via Gottardi 100, 41125 Modena, Italy

**Keywords:** COVID-19, Health policy, Data envelopment analysis, OECD

## Abstract

The COVID-19 pandemic is a global challenge to humankind. To improve the knowledge regarding relevant, efficient and effective COVID-19 measures in health policy, this paper applies a multi-criteria evaluation approach with population, health care, and economic datasets from 19 countries within the OECD. The comparative investigation was based on a Data Envelopment Analysis approach as an efficiency measurement method. Results indicate that on the one hand, factors like population size, population density, and country development stage, did not play a major role in successful pandemic management. On the other hand, pre-pandemic healthcare system policies were decisive. Healthcare systems with a primary care orientation and a high proportion of primary care doctors compared to specialists were found to be more efficient than systems with a medium level of resources that were partly financed through public funding and characterized by a high level of access regulation. Roughly two weeks after the introduction of ad hoc measures, e.g., lockdowns and quarantine policies, we did not observe a direct impact on country-level healthcare efficiency, while delayed lockdowns led to significantly lower efficiency levels during the first COVID-19 wave in 2020. From an economic perspective, strategies without general lockdowns were identified as a more efficient strategy than the full lockdown strategy. Additionally, governmental support of short-term work is promising. Improving the efficiency of COVID-19 countermeasures is crucial in saving as many lives as possible with limited resources.

## Introduction

The ongoing global COVID-19 pandemic is a challenge to humankind with a high death toll — more than 2.2 million persons lost their lives, with a large number of countries worldwide affected, and in excess of 102 million people had contracted the viral disease as of January 2021 (Johns Hopkins University 2021) [1, 2]. COVID-19 is an exogenous health threat that poses a particular challenge for health systems worldwide [9] because (1) vaccines do not yet apply any relevant influence, (2) forecasting the number and severity of infections is difficult, and (3) empirical evidence regarding the suitability of pharmaceutical (COVID-19 testing) or non-pharmaceutical interventions (travel restrictions, social distancing measures, partial or complete lockdowns) is missing [119]. As the pandemic is still ongoing, this poses a specific and comprehensive challenge to research and science to address this problem with all available tools, methods, and insights. The efficient allocations of health care resources, e.g., testing policies [27], hospital admissions [73], and intensive care capacity [132], are major health policy challenges [43, 55]. Therefore, an evaluation of the actions taken to date to address the pandemic is highly relevant for health economics [23, 72]. Of particular interest in the context of pandemics is the tension between medical efficacy and economic efficiency [69], which is increased by the quality of health care and the equitable use of health goods. When evaluating medical or health policy interventions during or after pandemic outbreaks, health economics analyses adopt different perspectives: (1) the perspective of health service providers (doctors, hospitals) studying, e.g., direct costs necessary to treat patients [22, 84], stockpiling of drugs [7, 63], or withholding effective novel antidotes [90], (2 patient-centered investigations on, e.g., consumer learning in vaccination decisions [88], (3) examinations through the lens of vaccine producers, e.g., analyzing the profit-maximizing capacity [53], and (4) studies evaluating the economic effect of pandemics, e.g., on companies through the lens of employee absences from work [40], effects on tourism and certain production sectors [108], and overall effects on a country’s economy [74, 77]. The objective of our study was to provide a country-specific efficiency evaluation of the fight against COVID-19 for 19 OECD countries while focusing on the role of pre-pandemic health care policy and its interconnection to ad hoc interventions (case 1 and case 2), as well as the impact of COVID-19 as an exogenous health threat to the country’s economy (case 3) [8]. We aspired to answer the following research questions: (1) "*How efficiently did OECD countries handle the COVID-19 outbreak?"* and *(2) "What are the reasons for efficient or less efficient COVID-19 handling?"* We employed a multi-factor evaluation approach based on data envelopment analysis (DEA) as a neutral efficiency measurement method to enable fact-based discussions and decision processes. DEA is an established and widely used method for efficiency measurement in healthcare management [71, 79, 81, 118, 135]. The evaluation approach integrates different levels of governmental decision-making and from a cross-country, also chosen by, e.g., Vogler and Fischer [128],: (1) factors considering pre-pandemic government health strategies measurable through governmental expenditures, (2) pre-pandemic characteristics of the respective health system representing health resources, (3) indicators expressing the consequences of governmental interventions during the COVID-19 outbreak on the economy, and (4) governmental interventions against COVID-19 taking into account the country-specific state of the pandemic. While our input factors are chosen to quantify the direct costs that are necessary for the treatment of a patient (health expenditures, number of doctors and hospital beds), the output factors are not quantified in monetary terms but as therapeutic outcomes and presented as clinical or physical quantities (infections, deaths, recoveries) because we wanted to consider patient-relevant measures.

The contribution of this paper is (1) the specific quantitative efficiency measurement approach with the DEA technique applied to a strategic-level evaluation of COVID-19 responses in the 19 examined OECD countries. In addition, this enables (2) a new perspective on COVID-19 countermeasures from an integrated health care and economics perspective based on empirical real-life data. Furthermore, (3) evaluations of individual measures directed toward the objective of an overall resource-efficient answer to viral pandemics that can be analyzed as a general objective measure are introduced. Therefore, we add new insights on the existing lessons learned from the management of the COVID-19 pandemic presented by Forman et al. [52].

This paper is structured as follows: the literature review section highlights the intersection of efficiency measurement and health economic activities during epidemics and pandemics from an interdisciplinary perspective (Sect. Literature view). Then, the methodology section describes the data used in this paper and elaborates on the use of DEA models to measure the efficiency of health care policies during the COVID-19 outbreak (Sect. 3). Section 4 presents the results of three longitudinal efficiency analyses (1) investigating the health system efficiency during COVID-19, (2) decompensating the health system and governmental ad hoc intervention efficiency, and (3) examining the impact of interventions on the country's economies. The results are discussed in Sect. 5. Finally, conclusions and the outlook toward future research are presented in Sect. 6.

### Literature review

A brief literature review was applied to structure a conceptual basis for the specific analysis implemented in this paper regarding COVID-19 overall efficiency evaluation. This is important as though it seems from the 2020 perspective that pandemic challenges are new on a global scale, this is not the case when looking back in a larger timeframe. In order to identify the key words and topics from previous research on this issue for the last 150 years, an overview is implemented as follows: For keywords, the following entries were used in searches of international academic journals: (1) “pandemic”, in combination with (2a) “public economics”, (2b) “government policy”, and (2c) “efficiency”. In a second search round, “pandemic” was replaced by “COVID-19”. Papers were selected to achieve a topical representation.

The structured results are presented in Table 1. Pandemic situations have been regarded as *rare but special public economic and health challenges* since the start of scientific discussions thereof in the nineteenth century (No 1–3 in Table 1). Additionally, characteristically, efficacy evaluation to date has occurred from *many different medical science perspectives*, including health care, public economics, and public health science as well as social sciences; discipline-specific journals are indicated in Table 1 (No 4–11). This pandemic is nevertheless seldom seen as a public economic challenge, as only very large pandemic situations actually strain public resources in the sense of crucial political and economic decisions to be made. Only rare events have a significant impact on public and private economic development in societies, and this is not yet reflected adequately in the research literature; this is another thing the current 2020 pandemic has changed. Finally, the COVID-19 pandemic in 2020 has already brought about a dedicated and enormous body of research literature in a broad range of disciplines, which corresponds with the global public economic impact of COVID-19 within this short timeframe (No 12–22). Case studies and different research results are reported from a very diverse set of countries as the COVID-19 pandemic has struck all countries (No 23–26). The research includes very specific questions, such as economic perspectives and business impact, engineering perspectives regarding safety impacts, and medical perspectives on cross-effects from other diseases and their treatment (No 27–32). Further interesting comparative analyses with earlier results can and will be addressed in future research with further insights. For the subsequent parts of this paper it is important to recognize that pandemic crises have been subject to research from many disciplines already — but without the methodology to integrate that into a coherent quantified evaluation scheme as it is proposed in this paper [18, 19].

**Table 1:** Literature analysis regarding pandemic situations from the public health perspectiveKlicken oder tippen Sie hier, um Text einzugeben

No	Author(s)	Year	Title	Journal	Perspective	Results
1	Radcliffe [107]	1862	On the recent epidemic of diphtheria	The Lancet	Medical Science	Not specified
2	(unknown)	1865	The cholera	The Lancet	Medical Science	Not specified
3	Sykes	1890	The influenza epidemic of 1889–1890	Public Health	Public Health	Fast dispersion of pandemic from Asia to Europe within weeks; comparison of influenza and dengue as north–south split
4	Kartman [75]	1957	The concept of vector efficiency in experimental studies of plague	Experimental Parasitology	Public Health	Mathematical models for epidemic modeling
5	Bloom and Mahal [18]	1997	Does the AIDS epidemic threaten economic growth?	Journal of Econometrics	Economics	Connection of epidemic events to economic progressions
6	Blount et al. [19]	1997	Nonlinear and dynamic programming for epidemic intervention	Applied Mathematics and Computation	Mathematics	Epidemics as case study examples for mathematical modeling
7	Mesnard and Seabright [91]	2009	Escaping epidemics through migration? Quarantine measures under incomplete information about infection risk	J. of Public Economics	Public Economics	Effectiveness of quarantine measures given incomplete personal information and the motivation/decision to embark on migration to evade individual infections
8	Dasaklis et al. [34]	2012	Epidemics control and logistics operations: A review	Int. J. of Production Economics	Management Science	Impact of epidemics on production and logistics environments and management
9	Naevdal [95]	2012	Fighting transient epidemics – optimal vaccination schedules before and after an outbreak	Health Economics	Public Economics	Showing increasing returns to scale for a vaccination with influenza as an example
10	Cao et al. [24]	2017	Global stability of an age-structure epidemic model with imperfect vaccination and relapse	Physica A: Statistical Mech.& its Applications	Mathematics	Modeling of interventions for global epidemic events
11	Kostova et al. [83]	2019	Long‐distance effects of epidemics: Assessing the link between the 2014 West Africa Ebola outbreak and U.S. exports and employment	Health Economics	Public Economics	Economic transfer effects of epidemic and pandemic events with the example of Africa and the USA from 2014
12	Zhai et al. [136]	2020	The epidemiology, diagnosis, and treatment of COVID-19	Int. J. of Antimicrobial Agents	Medical Science	Specific results regarding COVID-19 treatment from a medical perspective
13	Singh et al. [116]	2020	Internet of things (IoT) applications to fight against COVID-19 pandemic	Diabetes & Metabolic Syndrome	Medical Science, Computer Science	Expectations and results regarding IoT concepts and instruments anti-pandemic
14	Bontempi et al. [21]	2020	Understanding COVID-19 diffusion requires an interdisciplinary, multi-dimensional approach	Environmental Research	Environmental Science	The requirement of an interdisciplinary approach toward COVID-19 measures
15	da Silva et al. [33]	2020	Forecasting Brazilian and American COVID-19 cases based on artificial intelligence coupled with exogenous climatic variables	Chaos, Solitons & Fractals	Mathematics	Pandemic case prognosis with specific models, including temperature and weather data
16	Eberhardt et al. [42]	2020	Multi-stage group testing improves efficiency of large-scale COVID-19 screening	J. of Clinical Virology	Medical Science	Testing strategies regarding public policy and decision-making information in a pandemic
17	Govindan et al. [60]	2020	A decision support system for demand management in healthcare supply chains considering the epidemic outbreaks	Transportation Research Part E	Management Science	Stabilizing global supply chains in pandemic situations
18	Wang et al. [130]	2020	Psychological impact of Coronavirus Disease 2019 (COVID-19) epidemic on medical staff in different posts in China	J. of Psychiatric Research	Medical Science	Cross-disciplinary effects of pandemics on healthcare staff with the example of China
19	Yin et al. [134]	2020	Preventing COVID-19 from the perspective of industrial information integration	J. of Industrial Information Integration	Computer Science	Specific information and integration perspective on pandemic countermeasures
20	Wang et al. [129]	2020	Can masks be reused after hot water decontamination during the COVID-19 pandemic?	Engineering	Engineering Science	Operational question of protective gear reuse in a pandemic situation and with specific hygiene measures
21	Kierzkowski and Kisiel [78]	2020	Simulation model of security control lane operation in the state of the COVID-19 epidemic	J. of Air Transport Management	Management Science	Extension of pandemic situation management toward airport security management
22	Alberti and Faranda [1]	2020	On the uncertainty of real-time predictions of epidemic growth: A COVID-19 case study for China and Italy	Communications in Nonlinear Science and Numerical Simulation	Mathematics	Ex-post evaluation of simulation and prognosis approaches in a pandemic
23	Yezli and Khan [133]	2020	COVID-19 social distancing in the Kingdom of Saudi Arabia	Travel Medicine & Infectious Disease	Public Health	Measures evaluation with Saudi Arabia as the example
24	Kawashima et al. [76]	2020	The relationship between fever rate and telework implementation as a social distancing measure against the COVID-19 pandemic in Japan	Public Health	Public Health & Public Economics	Interrelation of COVID-19 outbreak and telework measures with Japan as the example
25	Saez et al. [113]	2020	Effectiveness of the measures to flatten the epidemic curve of COVID-19. The case of Spain	Science of The Total Environment	Public Economics	Effectiveness evaluation of COVID-19 measures with Spain as the example
26	Vicentini et al. [127]	2020	Early assessment of the impact of mitigation measures on the COVID-19 outbreak in Italy	Public Health	Public Health	Impact of mitigation and lockdown measures from with Italy as the example
27	WHO Working Group	2020	A minimal common outcome measure set for COVID-19 clinical research	The Lancet	Medical Science	Tackling the data collection and standardization challenge in the COVID-19 outbreak, a common dataset is proposed with three core elements
28	Eng Koon [43]	2020	The impact of sociocultural influences on the COVID-19 measures—Reflections from Singapore	J. of Pain and Symptom Management	Public Health	Health care systems react to external shocks and challenges differently based on their different socio-cultural backgrounds and values
29	Bruinen de Bruin et al. [23]	2020	Initial impacts of global risk mitigation measures taken during the combatting of the COVID-19 pandemic	Safety Science	Engineering	Empirical impacts of social distancing and lockdown measures on different public accident and injury areas
30	Dawoud [35]	2020	Emerging from the other end: Key measures for a successful COVID-19 lockdown exit strategy and the potential contribution of pharmacists	Research in Social and Administrative Pharmacy	Public Economics	Role of pharmacies, interrelation of political measures with medical results
31	Chilton et al. [30]	2020	Beyond COVID-19: How the ‘dismal science’ can prepare us for the future	Health Economics	Public Economics	Public welfare and balancing editorial and commentary about the trade-offs regarding health economics perspectives on COVID-19
32	Castaldo et al. [25]	2020	Safety and efficacy of amiodarone in a patient with COVID-19	J. of the Am. Coll. of Cardiology—Case Reports	Medical Science	Effects and safety of specific drug use in COVID-19 patients as a secondary challenge for medical treatments

## Data and methodology

### Dataset

The data used for the efficiency analysis were retrieved from several databases. First, general country-specific data, e.g., health spending, number of doctors and hospital beds, and unemployment rates, were obtained through database queries from the OECD database OECD Stat [100]. Second, the number of performed COVID-19 tests was gained through the United Nations Office for the Coordination of Humanitarian Affairs (OCHA) and downloaded on November 16, 2020 [96]. Third, the number of COVID-19 cases and deaths, as well as the number of recovered COVID-19 patients, was retrieved from the COVID-19 Dashboard by the Center for Systems Science and Engineering at Johns Hopkins University (JHU) [39] and downloaded on November 16, 2020. Fourth, to interpret the efficiency scores of each country, we use data on non-pharmaceutical governmental interventions from the Assessment Capacities Project [6], which had 14,848 database records on reported measures for 194 countries and the COVID-19 Government Response Stringency Index (GRSI) of the Oxford Covid-19 Government Response Tracker [62]. In total, our dataset includes 19 countries with mostly publicly financed healthcare systems and a minimum total population of two million inhabitants: Australia (AUS), Austria (AUT), Belgium (BEL), Canada (CAN), Czech Republic (CZE), Denmark (DEN), Finland (FIN), France (FRA), Germany (DEU), Ireland (IRE), Italy (ITA), Japan (JPN), Netherlands (NLD), Norway (NOW), Slovenia (SLN), South Korea (KOR), Spain (ESP), Sweden (SWE), and the United Kingdom (GBR).

### Data envelopment analysis

To calculate the efficiency of each country, this paper proposed a DEA model with an output-oriented ratio form under constant returns to scale (CRS). In general, DEA is a non-parametric optimization method of mathematical programming for measuring the relative efficiency of decision-making units (DMUs) that have multiple inputs and outputs. A basic model was introduced by Charnes, Cooper, and Rhodes [29] based on the Koopmans activity analysis concept [82] together with the publications of Debreu and Farrell dealing with radial efficiency measurement [36, 49]. The optimization method can be based on CRS in the CCR model [29] or variable returns on scale (VRS) in the BCC model [11], and each case has an input or output orientation. The linear program for the CCR model is [32], pp. 23–24):

1 $$\left( {LP_{0} } \right) max_{\mu , \vartheta } \theta = \mu_{1} \gamma_{1o} + ... + \mu_{s} \gamma_{so}$$

2 $$subject\, to \,\vartheta_{1} x_{1o} + ... + \vartheta_{m} x_{mo} = 1$$

3 $$\begin{gathered} \mu_{1} \gamma_{1j} + .... + \mu_{s} \gamma_{sj} \le \vartheta_{1} x_{1j} + \ldots + \vartheta_{m} x_{mj} \hfill \\ \left( {j = 1, \ldots , n} \right) \hfill \\ \end{gathered}$$

4 $$\vartheta_{1} , \vartheta_{2} , \ldots , \vartheta_{m} \ge 0$$

5 $$\mu_{1} , \mu_{2} , \ldots , \mu_{s} \ge 0$$

The basic idea is to calculate an efficiency frontier that is used as a best practice input–output combination for the underlying production scenario. A score of 1.0 indicates that a DMU is efficient and on the efficiency frontier, whereas the relative inefficiency of a DMU can be determined by measuring the distance between the individual DMU performance and the efficiency frontier. Measuring efficiency under the assumption of CRS is known as overall technical efficiency (OTE). This includes the determination of inefficiency/efficiency based on (1) the input/output transformation, meaning pure technical efficiency (PTE), as well as (2) the size of operations, meaning the scale efficiency (S.E.). DEA is a frequently applied methodology for public health question on several aggregation levels, e.g., regarding (1) hospitals [3, 4],D. C. [50, 51, 87], (2) the evaluation of healthcare reforms [44, 105, 106], (3) healthcare infrastructures [26], and (4) health care systems [94]. It has also been applied to evaluate the health care production of OECD countries [125, 126].

The traditional DEA methodology was extended in several directions: To evaluate the efficiency of panel data and enable time series efficiency measurement per DMU, Charnes et al. [28] proposed DEA window analysis. The dynamic perspective of this model treats the same DMU occurring in different periods as entirely different DMUs. The major benefit of its moving average method is that the number of DMUs increase, and dynamic changes of the efficiency per DMU can be evaluated. Furthermore, to disclose the black-box assumption of the traditional DEA model, Färe and Primont [48] and Färe [46] proposed Network DEA where a production process is decompensated in sub-technologies or nodes. The specification of these nodes enables the examination of input/output allocations as well as intermediate products, which represent the entire production process [47, 120].

### Design and application of the DEA model

The selection of applicable inputs and outputs, as well as the design of a suitable DEA model, is a well-known source of pitfalls within the DEA literature [41]. Researchers are often facing a choice between the (1) empirical quantitative data that are published, e.g., by governments or organizations, and (2) the restrictions of the DEA model concerning input/output selection. Therefore, we followed a four-step framework for DEA application proposed by Jain et al. [70]: (1) select applicable inputs and outputs for the subject of research, (2) validate the inputs and outputs, (3) select the applicable DEA model, and (4) execute the DEA model in step 4.

First, possible input and output factors must be directly related to government policy and health policy, as well as the COVID-19 outbreak, and can be separated into four groups: (1) factors considering pre-pandemic health strategies measurable through governmental expenditures or revenues, e.g., health or pharmaceutical spending; (2) pre-pandemic characteristics of the respective health systems representing resources that are available during an epidemic outbreak and measurable through the existing health infrastructure, e.g., number of doctors and nurses, number of medical and nursing graduates, number of hospital beds and intensive care beds; (3) indicators expressing the quantifiable economic consequences of governmental interventions during the COVID-19 outbreak, e.g., unemployment rates or trade volumes; and (4) governmental interventions against COVID-19 taking into account the country-specific state of the epidemic outbreak, e.g., number of COVID-19 tests performed, number of COVID-19 infections, number of COVID-19 deaths, or number of patients recovered from COVID-19.

Second, to validate the inputs and outputs through empirical data, we follow the steps proposed by Dyson et al. [41]. First, mixing indices and volume measures, as well as integrating percentages, can lead to distortions of the efficiency values. Since most data are available in the form of ratios, volume measures, e.g., total population, are excluded from the analysis or converted to ratios. Consequently, the DEA model is calculated by applying indices per inhabitant or per 1,000,000 inhabitants. Second, linked input/output values have to be avoided, e.g., considering the total number of tests performed and the number of tests per inhabitant leads to distortions of the efficiency scores. Third, a cross-correlation of the available factors has to be avoided. For instance, health spending per capita and pharmaceutical spending per capita (r = 0.68), as well as the number of nurses and doctors (r = 0.76), are highly correlated. To finish step two, we define the following input (I) and output (O) factors: I_1_ health expenditures in US dollars per inhabitant, I_2_ number of doctors per 1,000,000 inhabitants, I_3_ number of hospital beds per 1,000,000 inhabitants, I_4_ number of COVID-19 tests per 1,000,000 inhabitants, O_1_ number of COVID-19 deaths per 1,000,000 inhabitants, O_2_ number of patients recovered from COVID-19 per 1,000,000 inhabitants, O_3_ number of COVID-19 cases per 1,000,000 inhabitants, and O_4_ unemployed individuals per 1,000,000 inhabitants assigned to the labor force. Especially for I_1_ to I_3_, we are aware of the fact that the possible regional organization of health care systems is a key issue for the efficient handling of pandemics. E.g., hospitals traditionally organized to deliver patient-centric care are ill-equipped to deliver the type of community-focused care needed during a pandemic (For research regarding the further COVID-19-related development of public hospitals, the reader is referred to Rodríguez et al. [110]. But as most health care systems are highly decentralized, it is difficult to quantify that different regions tried various policy responses. As spatial doctor density or any index accounting for the local public health infrastructure and surveillance (e.g., the number of community doctors as compared to that of hospital specialists) is not available in cross-country databases, our approach is based on the currently available data aggregation level of countries. Appendix Table 4 summarizes the key attributes of the dataset by applying descriptive statistics, and a further correlation analysis proves that there is no linear statistical relationship between the applied input and output measures. Hereafter, we explain the variables in detail:

*I*_*1*_ *Health expenditures in US dollars per inhabitant* measures the final consumption of health care goods and services that are financed through a mix of financing arrangements, including government spending and compulsory health insurance, as well as voluntary health insurance and private funds, such as households' out-of-pocket payments, NGOs and private corporations [98]. This factor quantifies the pre-pandemic government health care policies determining the majority of the health care resources that are available during the COVID-19 pandemic (fixed).

*I*_*2*_* Number of doctors per 1,000,000 inhabitants*, which defines doctors as practicing doctors providing direct care to patients. Doctors are usually generalists who assume responsibility for the provision of continuing care to individuals and families or specialists, such as pediatricians, obstetricians, gynecologists, psychiatrists, medical specialists, and surgical specialists [97]. As doctors are an essential resource in fighting pandemics, the input factor expresses a major pre-pandemic variable that reflects the resources of a health system (fixed).

*I*_*3*_* Number of hospital beds per 1,000,000 inhabitants* quantifies the available resources for delivering health services to patients in hospitals in terms of the number of beds that are maintained, staffed, and available for use. It is the second measure to quantify the pre-pandemic health system resources available to fight against COVID-19 [99].

*I*_*4*_ *Number of COVID-19 tests per 1,000,000 inhabitants* includes diagnostic testing for COVID-19, which looks for the presence of the virus in specimens obtained from patients. The number of performed tests is reported by OCHA [96]. It is a factor that quantifies the nature and extent of ad hoc government policies against COVID-19 and enables its integration in an efficiency analysis. An even better fit to quantify this aspect could be the number of tests performed per day compared to the total number of tests that can be processed per day. However, as this capacity is mostly depending on highly decentralized lab capacity, it was not quantifiable in the course of this research [21, 25].

*O*_*1*_* Number of COVID-19 deaths per 1,000,000 inhabitants* expresses the mortality of the pandemic and is, therefore, one of the most important measures to quantify the burden of COVID-19. Countries throughout the world have reported very different case-fatality ratios, e.g., the number of deaths divided by the number of confirmed cases [39]. O_1_ solely includes the reported deaths associated with COVID-19. As DEA would value a large number of deaths as a large output and, therefore, as highly efficient, O_1_ is integrated as an undesirable output.

*O*_*2*_* Number of patients recovered from COVID-19 per 1,000,000 inhabitants* quantifies the number of individuals who successfully recovered from their COVID-19 infection and were discharged from hospitals and self-isolation facilities. As the exact date of recovery is unknown in most cases, the Robert Koch Institute and Johns Hopkins University use algorithms to estimate the number of recovered cases [39].

*O*_*3*_*Number of COVID-19 cases per 1,000,000 inhabitants* quantifies the number of individuals who are infected within an examined time period. Reducing the number of new COVID-19 cases will help countries prevent overloading of their health care systems. Because the main transmission pathway for COVID-19 is the respiratory absorption of virus-containing fluid particles that are produced during breathing, coughing, speaking, and sneezing, governmental restrictions include partial or total lockdown, traveling limitations, and social distancing restrictions. O_3_ can be used to integrate an evaluation regarding the suitability of timing, nature, and extent for ad hoc governmental measures [39]. As DEA would value a large number of cases as a large output and, therefore, as highly efficient, O_3_ is integrated as an undesirable output.

*O*_*4*_*Unemployed individuals per 1,000,000 inhabitants assigned to the labor force* are integrated into a separate model to measure the impact of governmental restrictions on the economy, as well as the success of governmental interventions through, e.g., tax reductions, monetary subsidies for short-term work, or loan programs. Unemployed inhabitants are people of working age who are without work, are available for work and have taken specific steps to find work. This indicator is measured in numbers of unemployed people per 1,000,000 inhabitants of the labor force. The labor force is defined as the total number of unemployed people plus those in employment [101]. Thus, inhabitants outside of the working age are excluded. O_4_ is integrated as an undesirable output [31, 33, 35].

In step three of the DEA application framework, before the execution of the computations in step four, the number of defined inputs and outputs can also provide another pitfall, as discussed by Dyson et al. [41], who proposes that the number of observation points must be at least 2 times the number of inputs times the number of outputs. With four inputs, four outputs, and 228 observation points (19 countries times 12 periods, each lasting one week), our model fulfills this criterion. Furthermore, DEA models can aspire to (1) maximize desirable outputs, (2) minimize undesirable outputs, (3) maximize desirable inputs, or (4) minimize normal inputs [64]. In the course of this paper, we use an output-oriented model. As the output-oriented model seeks to maximize outputs, O_1_, O_3_, and O_4_ are treated as undesirable outputs that have to be minimized [61, 114, 115].

To support the choice of available scale assumption, semi-parametric statistical tests, known as Banker's tests or tests of goodness of fit, were applied; H_0_ was the assumption of CRS against the alternative of H_1_ with VRS [10, 12]. For execution, we follow the example of Giokas et al. [58], assuming that *h*^*C*^ and *h*^*B*^ are the DEA inefficiency scores estimated from the BBC and CCR models. In the first hypothesis test (formula 1), *h* is assumed to follow the exponential distribution in the two models and evaluated relative to the critical value of the F-distribution with (2 N, 2 N) degrees of freedom, while in the second (formula 2), *h* is assumed to be half-normally distributed for both models and evaluated relative to the critical value of the F-distribution with (N, N) degrees of freedom [58], p. 1941).

6 $$T_{EX} = \frac{{\mathop \sum \nolimits_{j} \left( {h^{C} - 1} \right)}}{{\mathop \sum \nolimits_{j} \left( {h^{B} - 1} \right)}}$$

7 $$T_{HN} = \frac{{\mathop \sum \nolimits_{j} \left( {h^{C} - 1} \right)^{2} }}{{\mathop \sum \nolimits_{j} (h^{B} - 1)^{2} }}$$

The results of T_EX_ = 2.03 and T_HN_ = 4.11 indicate that the null hypothesis of constant returns to scale cannot be rejected at the 5% level of significance for an exponential distribution and normally distributed *h* score. Therefore, we applied the CCR model with CRS.

## Empirical results

### Comparability of health systems in time-delayed COVID-19 outbreaks

Being able to evaluate and compare the efficiency of healthcare systems during the COVID-19 pandemic requires considering two circumstances that need to be addressed before applying the evaluation framework elaborated in the previous chapter: (1) the comparability of the health systems of OECD countries, and (2) time-delayed COVID-19 outbreaks and their operationalization. Several dimensions of a healthcare system, e.g., the level and financing of resources, the role of key actors including the state and other societal or private actors, and access regulation, should be noted when comparing various countries [45]. To systematize country-specific characteristics of health systems, research approaches have developed typologies of health systems that aim to better characterize and categorize health systems for European (P. L. [50, 51] and OECD countries [20, 109, 121, 131]. Böhm et al. [20] applied the typology developed by Rothgang et al. [111] that distinguishes the three dimensions of (1) regulation, (2) financing, and (3) service provision, as well as the three types of actors, namely (1) the state, (2) societal actors, and (3) private actors. Wendt [131] proposed four types of healthcare systems that mainly focus on health expenditure with public financing and out-of-pocket payments by patients, as well as on the payment of general practitioners and access regulation. Reibling et al. [109] develop five typologies integrating the dimensions: (1) the level of resources of healthcare systems operationalized through expenditure on healthcare per capita and the number of general practitioners per thousand inhabitants, (2) the public–private mix operationalized through the share of public health expenditures of the total health expenditure, the share of out-of-pocket payments of the total health expenditure, and the payment of specialists; (3) access regulation, including whether individuals are required to register at a general practitioner and how a person can access specialist care; (4) primary care orientation operationalized through the ratio of general practitioners to specialists and the share of health expenditure on outpatient care of the total health expenditure, and (5) the performance measured by the level of achievement for goals in the prevention and the quality of care. In our analysis, we included all countries that were and categorized then into the following clusters: (1) the supply- and choice-oriented public types (AUS, AUT, BEL, CZE, DEU, FRA, IRL, SVN), (2) the performance- and primary-care-oriented public types (FIN, JPN, KOR, NOR, SWE), and (3) the regulation-oriented public types (CAN, DNK, ESP, GBR, ITA, NLD). The fourth cluster (4), the low-supply and low-performance mixed type, was excluded because the available data were suitable, not suitable, or sufficient for the purpose of our analysis. Therefore, we only compare public health systems and exclude the supply- and performance-oriented private types [42].

Time-delayed outbreaks with a varying number of infections lead to country-specific scenarios in the context of COVID-19. While (1) AUT, BEL, CZE, ESP, FIN, FRA, IRE, ITA, NLD, NOR, and KOR flattened the COVID-19 curve in terms of the number of new infections per day until July 1, 2020; (2) CAN, DEU, DNK, ISL, SVN lowered the number of new infections significantly but had a noteworthy number of new infections per day; (3) GBR and SWE had a constantly growing number of new COVID-19 infections since the first death; and (4) AUS and JPN started the second infection wave in July 2020. Being able to deal with these time lags requires the normalization of all countries to a starting time t_0_ [92]. Thus, we chose the date of the first COVID-19-related death reported by Johns Hopkins University as the beginning of the country-specific COVID-19 outbreak. With this normalization approach, we were able to compare the healthcare system efficiency for the selected OECD countries with the individual progression of COVID-19-related deaths and infections.

### DEA window analysis with panel data for time-dependent COVID-19 development

To evaluate the efficiency of health system policies before the epidemic outbreak, we separated the dataset into periods lasting seven days each and conducted a DEA window analysis. The first period started when the first COVID-19-related death appeared. We chose w = 2 as the window width for this analysis, as the maximum incubation time of the COVID-19 virus is 14 days, which is, on the other hand, the minimum period until the effects of governmental practices are measurable. The first model used I_1_, I_2_, and I_3_, as well as O_1_ and O_2_. Table 2 summarizes the results.

**Table 2:** Evaluation of country-specific efficiency through the lens of health system efficiency

	Mean	1	2	3	4	5	6	7	8	9	10	11	12
AUS	0.96	1.00	0.97	0.95	0.95	1.00	0.95	1.00	1.00	0.89	0.83	1.00	1.00
AUT	0.75	0.68	0.61	0.68	0.96	1.00	0.94	0.81	0.63	0.73	0.73	0.67	0.59
BEL	0.79	0.51	0.53	0.59	0.80	0.90	0.92	1.00	0.98	0.87	0.73	0.80	0.84
CAN	0.94	1.00	0.79	0.86	1.00	1.00	1.00	0.98	0.89	0.87	0.96	1.00	0.93
CZE	0.85	0.77	0.77	0.90	0.92	1.00	0.97	0.78	0.70	0.73	0.71	0.96	1.00
DEU	0.84	0.65	0.66	0.59	0.89	0.98	1.00	0.96	0.91	0.91	0.84	0.81	0.88
DNK	0.96	0.94	1.00	0.90	0.91	0.92	1.00	1.00	0.97	1.00	1.00	1.00	0.89
ESP	0.80	0.67	0.52	0.51	0.77	0.85	1.00	1.00	0.97	0.92	0.87	0.73	0.84
FIN	0.99	1.00	1.00	1.00	1.00	1.00	0.95	1.00	1.00	1.00	1.00	0.95	1.00
FRA	0.69	0.58	0.63	0.77	0.76	0.78	0.69	0.58	0.54	0.60	0.77	0.86	0.75
GBR	0.69	0.73	0.55	0.53	0.52	0.52	0.61	0.66	0.65	0.73	0.89	0.99	0.95
IRL	0.85	0.54	0.69	0.54	0.74	0.92	0.86	0.94	1.00	1.00	1.00	0.95	1.00
ITA	0.74	0.77	0.56	0.52	0.51	0.52	0.63	0.80	0.82	0.85	0.94	0.98	0.98
JPN	0.99	1.00	1.00	0.89	1.00	0.99	0.99	1.00	1.00	1.00	1.00	0.98	1.00
KOR	0.88	0.61	0.57	0.76	1.00	0.89	0.90	0.96	0.96	0.99	0.97	0.93	0.99
NLD	0.99	1.00	1.00	1.00	1.00	0.96	1.00	0.94	1.00	1.00	1.00	1.00	1.00
NOR	0.98	1.00	1.00	1.00	1.00	1.00	1.00	0.92	1.00	1.00	0.83	1.00	1.00
SVN	0.68	0.56	0.98	0.86	0.69	0.70	0.72	0.65	0.60	0.67	0.57	0.55	0.66
SWE	0.99	1.00	1.00	1.00	0.93	1.00	0.94	0.99	0.97	1.00	0.99	1.00	1.00

X Period of partial/full lockdown implementation

1. AUT: Two announcements for partial lockdowns were implemented on March 16, 2020, and March 19, 2020, which was at the end of period 1 in our analysis (Austrian Federal Ministry of Social Affairs, Health, Care and Consumer Protection, 2020). Two weeks later, the efficiency level of Austria increased significantly. After releasing the lockdown in period 6, several local COVID-19 outbreaks led to COVID-19-related death peaks and consequently to a low level of efficiency in subsequent periods.
2. BEL: An equal logic can be inferred in the case of Belgium, where a general lockdown starting on March 18, 2020 [15] limited the leaving of homes, except for emergencies, in period two of our analysis. At this time, the efficiency level was 0.53. Two weeks later, the efficiency level of Belgium increased significantly to 0.80 [59].
3. CZE: A nationwide quarantine was introduced from March 16, 2020 until April 11, 2020 (period 1), except for essential needs, helping others, and necessary trips to families (U.S. Embassy in the Czech Republic, 2020). Two weeks later, the efficiency level of CZE increased significantly from 0.77 in period 1 to 0.90 in period 3. The low-efficiency level in periods 7 to 10 results from a constant (low) death rate, while other countries manage to significantly reduce the number of new deaths in weeks 7 to 10 after the first death.
4. DEU: After a constant increase in new COVID-19 infections, Germany introduced a national lockdown in period 2 on March 22, 2020 [57]. Only essential trips, including for work, were allowed and Germany had more stringent public movement rules. The efficiency level increased significantly from 0.66 to 0.89 and remained constant until the end of the first COVID-19 infection wave.
5. ESP: Starting on March 16, 2020, the government only allowed movements for travels to make essential purchases (food, hygiene, health, first necessity), in strict compliance with basic precautionary measures, travels to access banking services, or travels to provide care and assistance to vulnerable people [117]. After introducing these measures in period 2, with an average efficiency of 0.52, positive development started in period 4 (eff. = 0.77). The decline in efficiency in periods 10 to 13 can be traced back to a high death rate in relation to other countries in these late periods. Therefore, the speed of flattening the curve had a significant impact on countries’ efficiency in fighting the pandemic.
6. FRA: One of the countries with the lowest efficiency level among all periods implemented the lockdown on March 17, 2020, and from a calendrical perspective, FRA had efficiency levels quite similar to those in other European countries [54]. However, France was already in period 5, meaning one month and two days after the first reported COVID-19-related death. It is furthermore interesting to observe that the late lockdown did not have the same effect as the early (period 1 or 2) lockdowns of other countries, as the efficiency level of France constantly remained low in subsequent periods (period 7 and further).
7. UK: The government implemented a lockdown on March 24, 2020 (end of period 3), which was late compared to more efficient European countries, e.g., Germany or Spain. Additionally, the health system has significantly fewer doctors per million inhabitants and hospital beds per million inhabitants compared to Italy and France, which have similar populations. Finally, the resulting constantly high death ratio, with 500 to 1,100 deaths per day during April, as well as high death ratios in the late periods (compared to other countries), led to an overall weak health system performance [60, 66].
8. IRE: The government enforced a partial lockdown on March 23, 2020 (period 2); people could only leave their homes to travel to or from work if they were providing an essential service, to shop for food, to collect medical prescriptions and medical supplies and attend medical appointments, or to carry out vital services, such as caring for a family member, within 2 kms of their houses, while keeping 2 m away from others for social distancing. Similar to other states, the results were observable after two periods in period 4 [67]. Ireland’s health system and additional measures made it possible to maintain a high efficiency level for the rest of the pandemic.
9. ITA: In Italy as one of the worst affected countries during the first COVID-19 wave the political measures developed as follows: On March 8, a decree approved by the Council of Ministers introduced quarantine measures in northern Italy. The regions Lombardy, Veneto, Emilia Romagna and Piedmont are affected and 15 million people — about a quarter of the Italian population – are confined. On March 9, this lockdown was extended to the whole country. All public spots were closed; travel was limited to the strictly necessary (health, food, and work when working remotely is impossible). All gatherings were prohibited and punishable by fines. On March 11, adoption of a new decree from the Council of Ministers imposed the closure of all businesses except for pharmacies and grocery stores. March 11 was therefore basically the start of the nation-wide lockdown. On March 22 and 23, this was de jure adopted by an ordinance by the Ministries of Health and of the Interior prohibiting citizens from leaving their city of lockdown, but this simply did not add anything at all to March 11 decree [68]. About three weeks later, the efficiency level in Italy increased significantly. However, the low average efficiency of Italy can be connected to the fact that the lockdown was implemented late when comparing with the timing in other states. The same observation was made for Great Britain [75, 76, 78]. For a deeper discussion on the measures taken by the Italian government, the reader is referred to Mauro and Giancotti [89] and Valent et al. [124].
10. KOR: Until July 2020, South Korea was the only country with a population of over 50 million inhabitants that had slowed the spread of the virus and flattened the curve of new infections without shutting down the whole country. Furthermore, there were no extreme personal travel or movement restrictions and no closure of airports. After the first COVID-19 case on January 19, 2020, and the first COVID-19-related death on February 20, 2020, South Korea raised the alert level to red on February 23, 2020 (period 1) (The Government of the Republic of Korea, 2020). The country managed to avoid high case numbers through intelligent and digital COVID-19 management, as well as persistent tracking, tracing, and testing of infected persons who were quickly identified and treated at an early stage. The country is thus a unique example of efficient COVID-19 control without lockdown. However, in many European and North American countries, involving police in matters of public health combined together with data sharing is seen as an act of criminalizing illnesses and, therefore, highly critical [93].
11. SVN: The country with the least inhabitants in the whole dataset also had the lowest input values for the average health spending per capita. On the other hand, the number of doctors per million inhabitants and the number of hospital beds per million inhabitants are higher in better-developed countries, e.g., Belgium, Great Britain, the Netherlands, or Norway. Due to the number of inhabitants (2 million), it is difficult for Slovenia to find measures that keep the relative number of deaths or new cases of infections as low as for countries with more than 50 times more inhabitants (e.g., Japan). Therefore, Slovenia can be seen as an example for the case that fewer infections should have been possible through a theoretic performance-oriented view, while this seems unrealistic from a practical viewpoint [86].
12. AUS, CAN, JPN, and NLD have early lockdowns in the first period and consequent infection tracking in common, which led to a stable and, compared to other countries, highly efficient health system with a mean efficiency score above 0.90.
13. Similar policies were adopted in the Northern European countries of DNK, FIN, NOR, and SWE, to keep restaurants and primary schools open and rely on citizens adhering to social distancing recommendations themselves. This seems to be another highly successful strategy, as the mean efficiency score was constantly above 0.90. Furthermore, these states have highly developed public health systems.

### Network DEA for pre-epidemic health strategy and COVID-19 testing as an ad hoc intervention

**Fig. 1:**
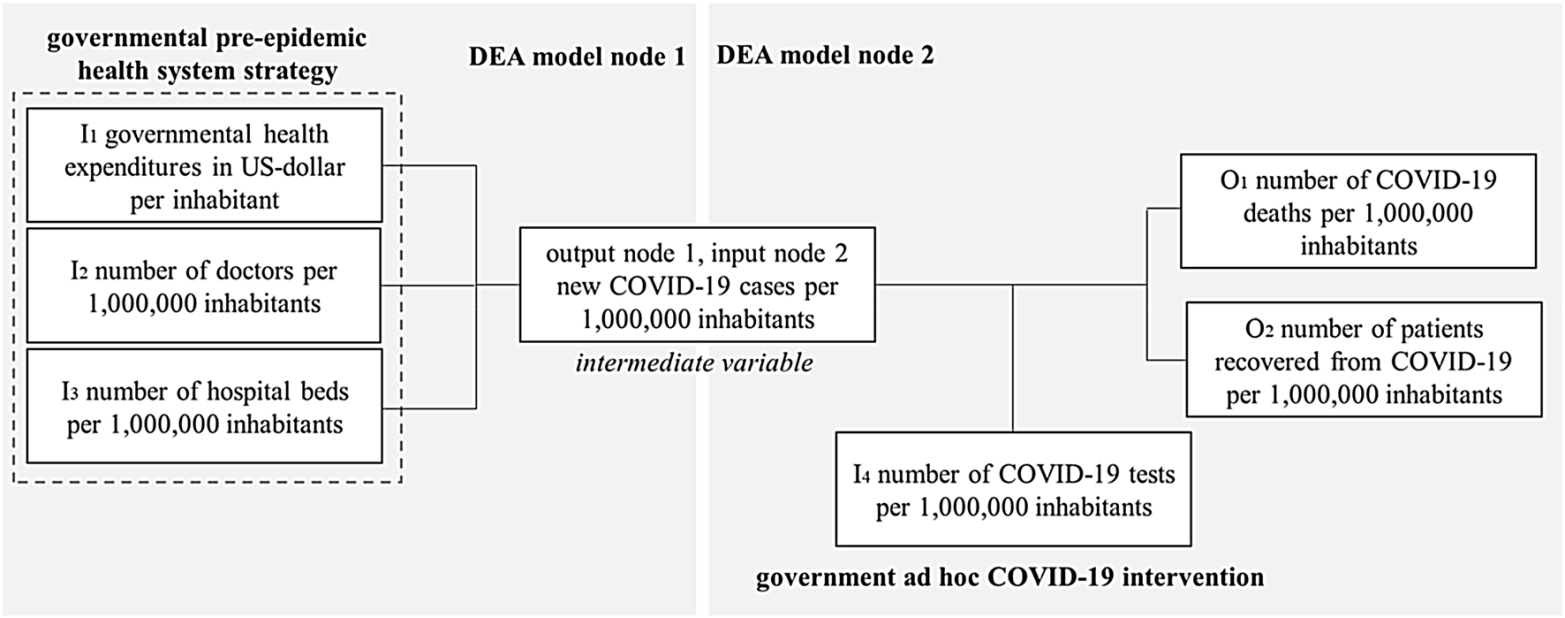
Network DEA with pre-epidemic health strategy and COVID-19 testing ad hoc intervention

To determine more about the interdependency of pre-epidemic health system strategies and ad hoc interventions when fighting COVID-19, the following network DEA approach separates the efficiencies of both contributing factors. Figure 1 summarizes the approach [95].

**Fig. 2:**
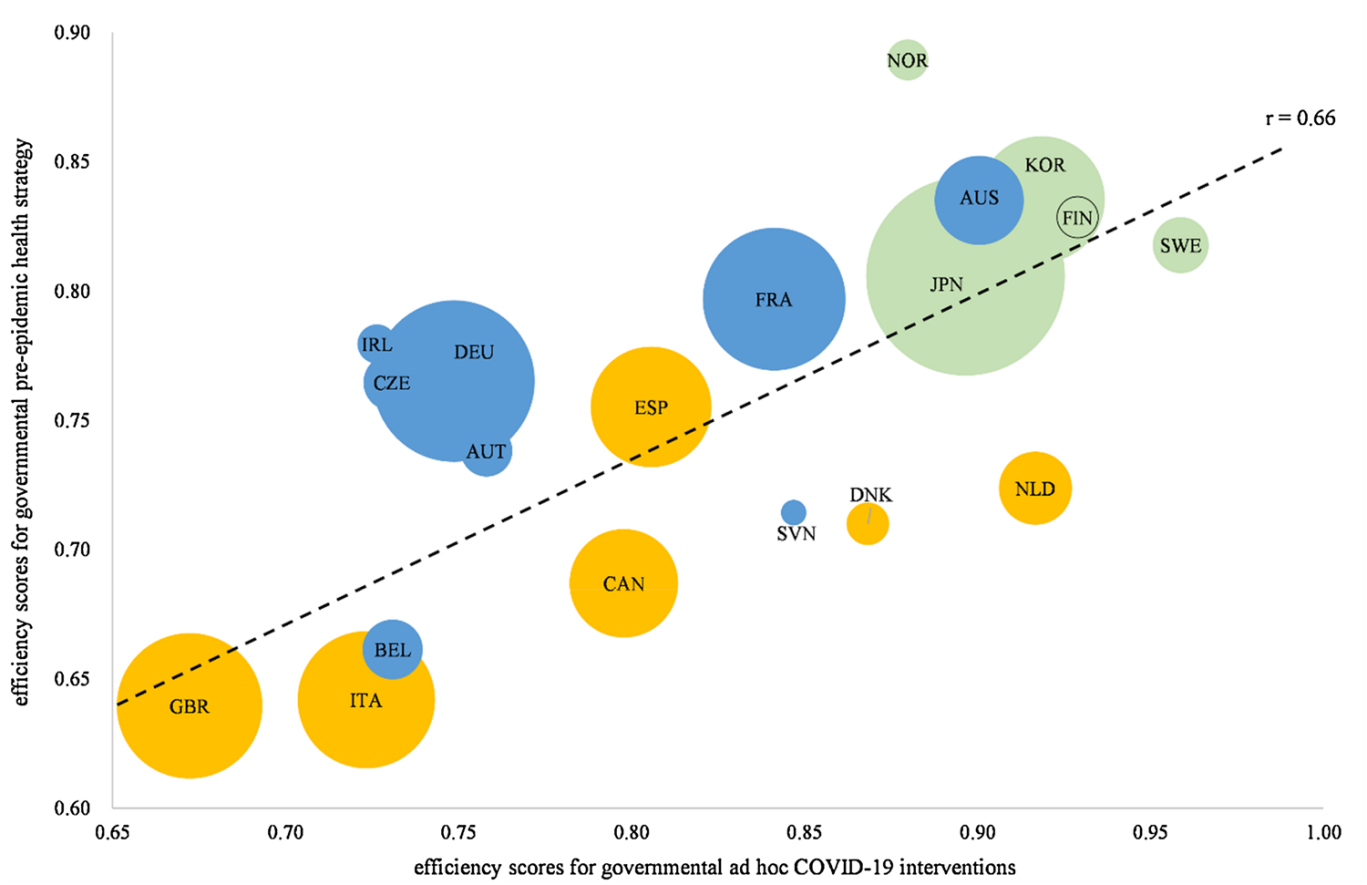
Relationship of efficiency for pre-epidemic health strategy and COVID-19 testing policy

The results of the network DEA (appendices 2 and 3) indicate that the efficiency of the pre-epidemic health strategy and ad hoc COVID-19 intervention are positively related to each other (r = 0.66). This seems reasonable when remembering the example of Germany expanding the intensive care hospital capacity based on the existing health care system capacity. Furthermore, the possibility of testing for COVID-19 infections depends on the available health care resources, e.g., doctors or medical professionals. Therefore, an important finding of the network DEA is that the institution of only ad hoc decisions, e.g., lockdowns, testing, or expanding intensive care capacity, will not lead to an overall successful fight against COVID-19. Rather, ad hoc decisions can contribute to the fight against COVID-19 and increase efficiency. Figure 2 demonstrates the relationship between efficiency for pre-epidemic health strategy and COVID-19 testing policy, where the size of the bubbles depends on the population of the country and the color indicates the membership to one of the examined healthcare system typologies: (1) the supply- and choice-oriented public type (blue), (2) the performance- and primary-care-oriented public type (green), (3) the regulation-oriented public type (yellow).

Through a closer examination of the efficiency values per typology, it can be stated that performance- and primary-care-oriented public healthcare systems have been the most efficient ones in fighting the COVID-19 pandemic. This is true for the pre-pandemic health system efficiency, as well as for the efficiency of ad hoc measures. According to Reibling et al. [109], this type is dominated by public financing but spends less money and uses fewer human resources to provide healthcare. The focus of the FIN, JPN, KOR, NOR, and SWE healthcare systems was clearly on primary care orientation, with a comparatively high proportion of primary care doctors compared with specialists [107, 108].

Furthermore, the regulation-oriented public type was the least efficient in regard to the efficiency of the pre-pandemic healthcare strategy. This type was characterized by a medium level of resources that come primarily through public funding, but it has the highest level of access regulation and limits the choice of providers. The system is also characterized by the absence of formalized cost-sharing and the lowest level of out-of-pocket expenditures [109]. It is important to mention that countries in the regulation-oriented public type can increase their overall performance in fighting COVID-19 with good ad hoc management, e.g., through lockdowns or social distancing policies (DNK, NLD). However, incorrect decisions such as lockdowns that are introduced late (GBR) or only in small steps and inconsistently (ITA), together with regulation-oriented or inefficient public pre-pandemic healthcare system policies, led to the lowest efficiency levels among all OECD countries. A more counter-intuitive finding is that the efficiency was not dependent on the population size of the countries (node 1: r =– 0.12; node 2: r =– 0.06), population density (node 1: r =– 0.01; node 2: r =– 0.24), median age (node 1: r =– 0.11; node 2: r =– 0.26), proportion of the population that is older than 65 years (node 1: r = 0.03; node 2: r =– 0.17), the proportion of the population that is older than 70 years (node 1: r =– 0.08; node 2: r =– 0.23), the GDP per capita (node 1: r = 0.05; node 2: r = 0.33), or the Human Development Index (node 1: r =– 0.14; node 2: r =– 0.11) [123].

### DEA window analysis for the impact of governmental programs on the economy

Governmental regulations to lower the spread of COVID-19 through, e.g., isolation and quarantine policies, public gatherings limitations, or lockdowns, had versatile impacts on the economy. While the northern European countries Sweden (Government Response Stringency Index, GRSI 46.3 of 100) and Finland (GRSI 57.41 of 100) were defensive in their regulation policies compared to, e.g., France (GRSI 90.74 of 100) or Germany (GRSI 60.65 of 100). Therefore, the question of which policy is the best for a country’s economy arises [62]. A second important factor is a direct intervention through, e.g., limiting product imports/exports and government aid programs. Table 3 presents the results of the DEA window analysis using I_1_, I_2_, and I_3_, as well as O_1_, O_2_, and O_4_.

**Table 3:** Evaluation of country-specific efficiency through the lens of economic efficiency

	Mean	1	2	3	4	5	6	7	8	9	10	11	12
AUS	0.98	1.00	0.99	0.99	0.99	1.00	0.97	1.00	1.00	0.92	0.87	1.00	1.00
AUT	0.87	1.00	0.86	0.77	0.96	1.00	0.95	0.89	0.74	0.88	0.88	0.77	0.72
BEL	1.00	1.00	1.00	1.00	1.00	1.00	1.00	1.00	1.00	1.00	1.00	1.00	1.00
CAN	0.95	1.00	0.85	0.90	1.00	1.00	1.00	0.98	0.89	0.87	0.96	1.00	0.93
CZE	1.00	1.00	1.00	1.00	1.00	1.00	1.00	1.00	1.00	1.00	1.00	1.00	1.00
DEU	0.89	1.00	0.83	0.69	0.89	0.98	1.00	0.96	0.91	0.91	0.84	0.81	0.88
DNK	1.00	1.00	1.00	0.99	1.00	1.00	1.00	1.00	1.00	1.00	1.00	1.00	0.96
ESP	0.81	0.67	0.52	0.51	0.77	0.87	1.00	1.00	0.98	0.93	0.89	0.75	0.85
FIN	1.00	1.00	1.00	1.00	1.00	1.00	0.98	1.00	1.00	1.00	1.00	1.00	1.00
FRA	0.81	0.92	1.00	0.98	0.95	0.93	0.77	0.60	0.57	0.61	0.80	0.88	0.77
GBR	0.72	0.93	0.59	0.56	0.55	0.54	0.61	0.66	0.65	0.73	0.89	0.99	0.95
IRL	0.94	0.84	0.90	0.83	0.87	0.96	0.95	0.99	1.00	1.00	1.00	0.98	1.00
ITA	0.76	0.78	0.57	0.53	0.52	0.52	0.64	0.83	0.87	0.89	0.96	0.98	0.98
JPN	1.00	1.00	1.00	1.00	1.00	1.00	1.00	1.00	1.00	1.00	1.00	1.00	1.00
KOR	1.00	1.00	1.00	1.00	1.00	1.00	1.00	1.00	1.00	1.00	1.00	0.95	1.00
NLD	1.00	1.00	1.00	1.00	1.00	1.00	1.00	1.00	1.00	1.00	1.00	1.00	1.00
NOR	1.00	1.00	1.00	1.00	1.00	1.00	1.00	1.00	1.00	1.00	1.00	1.00	1.00
SVN	1.00	1.00	1.00	1.00	1.00	1.00	1.00	1.00	1.00	1.00	1.00	1.00	1.00
SWE	1.00	1.00	1.00	1.00	1.00	1.00	1.00	1.00	1.00	1.00	1.00	1.00	1.00

Furthermore, the average efficiency level in the economic model was correlated with the average GRSI between periods 1 and 12 per country. Taking the averages for the whole dataset (periods 1–12) showed a negative correlation of efficiency and GRSI (r =– 0.45); the average of periods 5–12 showed a strong negative relationship, with r =– 0.54, and no significant relation for the last periods (10–2; r =– 0.28). For all moving average calculations, the correlation coefficient was negative (Appendix Table 7).

Summarizing these findings from an economic point of view, the higher the strength of the governmental response to COVID-19, e.g., through lockdowns and social distancing, the less efficient the country response is through the lens of economic efficiency. As presented in the previous chapters, lockdowns lead to a direct increase in efficiency, but the price is more unemployment and a weakening of the economy. The most promising approach to respond to COVID-19-related lockdowns of the economy and society is short-term work with governmental support, which had a positive impact on the efficiency observed the top 4 countries of Sweden (eff. = 1.00), Germany (eff. = 0.97), Spain (eff. = 0.93), as well as Switzerland (eff. = 0.84)^1^ and was not reported for the rest of the examined OECD countries [113, 116].

## Discussion

The following items for discussion stem from the presented results and are important for the objective of improving the public health economic answer to pandemic crises in a political and managerial sense:

1. Traditional health systems and economic success factors for wealth and development, such as country and population size or the respective Human Development Index (HDI), are found to be less relevant to the efficiency of pandemic management in some countries. In contrast, we have found that small and large countries, Eastern and Western counties, and developed and less developed are represented in both the efficient group and the less efficient group in terms of their efforts and results against the COVID-19 pandemic. This is important for health economics research and decisions, as new predictor variables for decisions and analysis regarding the resource-efficient answer to pandemic situations have to be identified and applied in future (e.g., the discussion by [30] or in a pre-COVID-19 perspective the results by [83] and [34]).
2. It is important to note that the development situation during a pandemic crisis is very dynamic, and individual countries are transitioning between various phases of success and failure at different times – successful crisis management in the past does not guarantee resistance to the pandemic for the future. This has already been found before the COVID-19 pandemic but has to be remembered nevertheless [24, 91]. In some cases, even a reciprocal connection can be recognized as successful crisis management is, by and large, lowering the acceptance of future restrictive measures in the fight against COVID-19. This is not only relevant to the ongoing discussions about “second waves” of COVID-19 but also the general risk of a long-term perspective and trade-off regarding health economic decisions. Research has to improve the analytical and prognosis perspective to enhance the pandemic foresight aspect of health economics. This also addresses a continuous learning perspective for the health care management discipline [65].
3. Looking at the integrated model of health care management and economic evaluation of country-specific crisis management provides insight that governmental support of short-term work is an efficient concept to balance COVID-19 intervention and economic success. This might be true for further instruments developed during the pandemic in 2020, including the latest measures taken by China, the U.S. and the European Union countries. Further methods and research discourse are required to systematize and evaluate all the comprehensive approaches regarding analysis and policies in this regard; in many cases, only long-term hindsight might reveal the full picture, as we have learned from previous pandemics (Almond & Bhashkar Mazumder, 2005 for the Spanish flu of 1918/19) [122, 127, 129].
4. Analysis and decision endeavors in health care management science have to take into account that international pandemic situations are real “rule changers” — many assumptions applied in health economics analysis and discourse simply do not hold in such situations, as already shown by Beutels et al. [17]. Therefore, dynamic and “out of the box” approaches have to be developed for application in situations that are beyond the routine steady state of health systems economics on normal days [85].
5. For further discussion regarding successful COVID-19 interventions, it may be beneficial to integrate data from deeper aggregation levels. An example is the impact of population size which has been found to be influential comparing rural and urban areas in Turkey [13], but had no impact on in our country-related analysis. The same applied for approaches that seek to evaluate regional changes of peoples’ actions, such as a more strict adoption of social distancing measures among the population, that reduce the spread [14]. However, as our research was intended to provide a bigger picture going beyond regional developments, the data quality on this aggregation level does not exist for a majority of OECD countries until now. The country managed to avoid high case numbers through intelligent and digital COVID-19 management, as well as persistent tracking, tracing, and testing of infected persons who were quickly identified and treated at an early stage. The country is thus a unique example of efficient COVID-19 control without lockdown.
6. The implementation of telemedical health services can be a promising concept for people seeking for a rapid first advice regarding a possible infection while keeping to social distancing measures at the same time. It could also help relieve pressure on the often overburdened primary care systems or emergency departments [56]. Parsons and Romanis [104] discusses an interesting case of a mobile abortion care service for the United Kingdom [103].
7. During our analysis, we found that all countries chose diverse strategies that are mostly disconnected. This applies for the OECD countries, as well as when looking at European countries in isolation (see also, [112]. The paradigms of strict legally backed lockdowns in central Europe stand against softer responses of northern European countries. Therefore, we agree with Forman et al. [52] and call for a unified responses to pandemics.
8. Additionally, our results on OECD countries may be supplemented by integrating developing OECD countries that we did not evaluate within the DEA model, e.g., (a) Chile, (b) Columbia, and (c) Mexico: We start with (a) Chile and the results presented by Oliveira et al. [102] showing that timely and coordinated social distancing was a powerful non-pharmaceutical intervention, but difficulties in keeping the population under control decreased the impact of this measure. For (a) Chile and (b) Columbia, Benítez et al. [16] show that although stringent measures of containment and mitigation were introduced, pre-pandemic conditions, e.g., high informal employment and social inequalities, have undermined the effectiveness of the countries’ responses to the pandemic. For (c) Mexico, Knaul et al. [80] demonstrate the absence of a uniform national response of Mexico and argue that coordinated, timely, rigorous response to the pandemic did not occur in Mexico. Arellanos-Soto et al. [5] evaluated Mexican Governmental measures by comparing data on influenza diagnosis, finding that the implementation of public health measures leads to a significant decrease. This is supported by Díaz-Castro et al. [38], concluding that Mexico's health policies had an effect on slowing the pandemic’s propagation, but population density and mobility played a fundamental role. In summary, the results for developing OECD countries are in line with our previously stated quantitative findings, including the suitability of social distancing measured and the fact that a lack of uniform, coordinated, and timely response may be reasons for countries to fall behind in an early stage. Furthermore, the cases of (a) Chile and (b) Columbia underline the relevance of pre-pandemic conditions [130, 133, 134, 136].
  Altogether, this approach, involving country-specific modeling of input and output types for a health and general economic efficiency analysis to be used as a central evaluation measurement for bundles of health care and political measures, has proven to be worthwhile and should be subject to further research and discussion. Further research approaches are warranted regarding, for example, the identification of further relevant input and output types, as well as the inclusion of further individual countries or bundles of countries besides the OECD set applied here. Additionally, as we identified (partial) lockdowns and social distance measures as a possible key to fight the pandemic, more research on the evolvement of public values and perceptions during the COVID-19 pandemic should foster our understanding on the acceptance level of these measures as discussed by Denburg et al. [37].

## Conclusion

This paper has outlined specific approaches and results regarding the efficiency of health economic measures and interventions for the COVID-19 pandemic worldwide, including country-specific analysis of 19 OECD countries. The results show that country-specific efficiency regarding multiple input and output factors varies very broadly and is not connected to traditional clustering factors or economic success factors, such as size, population, or development status. Instead, specific individual types of measures like selected lockdown or dedicated economic support instruments seem to make a difference, which is an important message for health economic research and management. Looking into further analyses based on these finding presented here will pay off in future as government interventions can be improved and finetuned to optimize the resource-dependent results from health policy-related answers to the COVID-19 crisis. The contribution of this paper consists of (1) the specific identification of efficient countries in the fight against the COVID-19 pandemic during the first waves in 2020, which can serve as role models and sources of further information through research into comparative efficiency the specific measures taken in these countries; (2) the establishment of an evaluation scale through multidimensional efficiency measures that provides room for further elaboration in health economic research to identify success factors; and (3) the introduction of specific political measures that are expected to be useful in many future situations similar to the COVID-19 situation. Therefore, the paper provides a valuable contribution toward an improvement regarding public measures for pandemic preparation and management.

## Appendix

See Appendix Tables 4, 5, 6 and 7.

**Table 4:** Attributes of the dataset and correlation matrix for applied input and output measures

	I1	I2	I3	I4	O1	O2	O3	O4
Min	2,057	0.002	0.002				0.010	0.046
Mean	3,877	0.004	0.005	0.005	11.950	117.626	139.191	1.039
Max	5,673	0.010	0.013	0.040	161.042	1,034.564	1,130.754	3.846
I1	1.00	0.130	– 0.130	0.130	–	0.080	0.070	– 0.220
I2		1.00	– 0.26	0.10	– 0.10	– 0.03	– 0.05	– 0.26
I3			1.00	– 0.32	– 0.21	– 0.26	– 0.28	0.09
I4				1.00	– 0.11	0.03	– 0.07	– 0.15
O1					1.00	0.70	0.78	0.14
O2						1.00	0.69	0.05
O3							1.00	0.07
O4								1.00

**Table 5:** Results of DEA network analysis per country and node with population

Country	Node 1	Node 2	Population
AUS	0.90	0.84	25,499,881
AUT	0.76	0.74	9,006,400
BEL	0.73	0.66	11,589,616
CAN	0.80	0.69	37,742,157
CZE	0.73	0.76	10,708,982
DEU	0.75	0.77	83,783,945
DNK	0.87	0.71	5,792,203
ESP	0.81	0.76	46,754,783
FIN	0.93	0.83	5,540,718
FRA	0.84	0.80	65,273,512
GBR	0.67	0.64	67,886,004
IRL	0.73	0.78	4,937,796
ITA	0.72	0.64	60,461,828
JPN	0.90	0.81	126,476,458
KOR	0.92	0.84	51,269,183
NLD	0.92	0.72	17,134,873
NOR	0.88	0.89	5,421,242
SVN	0.85	0.71	2,078,932
SWE	0.96	0.82	10,099,270
R	0.66	– 0.12	– 0.06

**Table 6:** Results of DEA network analysis per country, node and period

Period	Mean	1	2	3	4	5	6	7	8	9	10	11	12
*Node 1*													
AUS	0.90	1.00	0.79	0.78	1.00	0.70	0.81	1.00	1.00	1.00	0.94	0.92	0.87
AUT	0.76	0.55	0.54	0.61	0.62	1.00	1.00	0.85	0.87	0.68	0.72	0.78	0.88
BEL	0.73	0.50	0.54	0.53	0.58	0.69	0.74	0.82	0.87	1.00	0.85	0.85	0.80
CAN	0.80	1.00	0.77	0.78	0.81	0.91	0.99	0.78	0.69	0.70	0.76	0.69	0.68
CZE	0.73	0.55	0.63	0.80	0.78	0.83	0.84	0.84	0.68	0.58	0.69	0.74	0.81
DEU	0.75	0.56	0.57	0.55	0.55	0.62	0.89	0.86	0.81	0.90	0.88	0.90	0.89
DNK	0.87	0.64	0.99	0.70	0.66	0.88	0.97	0.93	0.90	0.87	0.93	0.98	0.97
ESP	0.81	0.60	0.52	0.52	0.63	0.84	1.00	1.00	0.90	1.00	0.96	0.83	0.86
FIN	0.93	1.00	0.90	0.91	0.99	0.95	0.95	0.94	0.87	0.87	1.00	0.85	0.91
FRA	0.84	1.00	1.00	0.75	0.77	0.79	0.67	0.59	0.70	0.88	0.97	1.00	0.98
GBR	0.67	0.69	0.59	0.63	0.60	0.57	0.63	0.71	0.68	0.71	0.78	0.75	0.73
IRL	0.73	0.54	0.53	0.58	0.58	0.55	0.56	0.69	0.84	1.00	0.92	0.94	1.00
ITA	0.72	1.00	0.63	0.56	0.53	0.53	0.61	0.75	0.76	0.77	0.82	0.85	0.86
JPN	0.90	1.00	1.00	0.88	0.84	0.84	1.00	0.83	0.81	0.83	0.86	0.87	1.00
KOR	0.92	1.00	0.76	0.80	1.00	1.00	0.95	0.82	0.86	1.00	1.00	1.00	0.84
NLD	0.92	1.00	0.79	0.80	0.84	0.98	0.92	0.88	0.88	1.00	1.00	0.97	0.95
NOR	0.88	0.57	0.85	0.81	0.67	1.00	1.00	0.87	1.00	0.86	0.93	1.00	1.00
SVN	0.85	0.52	0.94	0.72	0.71	0.90	0.92	0.89	0.93	1.00	1.00	0.90	0.73
SWE	0.96	1.00	0.92	0.89	0.89	0.93	1.00	0.93	0.98	1.00	0.97	1.00	1.00
*Node 2*													
AUS	0.84	0.51	0.55	0.90	1.00	0.79	0.92	1.00	0.98	0.95	0.72	0.88	0.83
AUT	0.74	0.61	0.62	0.60	0.86	1.00	1.00	0.84	0.60	0.77	0.66	0.63	0.66
BEL	0.66	0.51	0.55	0.52	0.54	0.60	0.63	0.73	0.92	0.80	0.65	0.75	0.73
CAN	0.69	0.58	0.52	0.55	0.54	0.67	0.76	0.72	0.68	0.70	0.78	0.98	0.77
CZE	0.76	0.93	0.96	0.79	0.70	0.69	0.68	0.59	0.60	0.73	0.66	0.87	0.98
DEU	0.77	0.64	0.65	0.56	0.58	0.78	0.93	1.00	0.93	0.94	0.74	0.70	0.72
DNK	0.71	0.51	1.00	0.63	0.52	0.68	0.78	0.61	0.65	0.82	0.92	0.77	0.64
ESP	0.76	0.50	0.50	0.53	0.67	0.86	1.00	1.00	0.98	0.90	0.78	0.66	0.68
FIN	0.83	0.95	0.88	0.80	0.61	0.63	0.64	0.81	0.82	0.94	1.00	0.90	0.96
FRA	0.80	1.00	1.00	0.53	0.52	0.78	0.67	0.64	0.61	0.90	0.92	1.00	0.99
GBR	0.64	0.50	0.51	0.54	0.58	0.52	0.58	0.67	0.69	0.69	0.78	0.86	0.75
IRL	0.78	0.50	0.62	0.53	0.60	0.58	0.67	0.90	0.98	1.00	1.00	0.99	1.00
ITA	0.64	0.50	0.50	0.50	0.50	0.51	0.59	0.76	0.78	0.71	0.76	0.82	0.77
JPN	0.81	0.99	1.00	0.58	0.64	0.54	0.68	0.72	0.73	0.90	0.97	0.93	1.00
KOR	0.74	0.50	0.50	0.70	1.00	1.00	0.61	0.60	0.74	0.93	0.97	0.71	0.56
NLD	0.72	0.50	0.50	0.51	0.51	0.58	0.73	0.77	0.91	1.00	0.96	0.90	0.82
NOR	0.89	0.72	0.92	0.86	0.88	1.00	0.95	0.72	0.87	0.94	0.86	1.00	0.95
SVN	0.71	0.59	1.00	0.76	0.62	0.64	0.60	0.54	0.70	0.96	1.00	0.64	0.54
SWE	0.82	0.50	0.64	0.82	0.66	0.67	0.71	0.89	0.97	1.00	0.97	0.99	1.00

**Table 7:** Results of the economics DEA model and Oxford COVID-19 Government Response Tracker

	1–12	2–12	3–12	4–12	5–12	6–12	7–12	8–12	9–12	10–12	11–12
*DEA model 3 efficiency scores*											
AUS	0.98	0.98	0.97	0.97	0.97	0.97	0.96	0.96	0.95	0.96	1.00
AUT	0.87	0.86	0.86	0.87	0.85	0.83	0.81	0.80	0.81	0.79	0.75
BEL	1.00	1.00	1.00	1.00	1.00	1.00	1.00	1.00	1.00	1.00	1.00
CAN	0.95	0.94	0.95	0.96	0.95	0.95	0.94	0.93	0.94	0.96	0.96
CZE	1.00	1.00	1.00	1.00	1.00	1.00	1.00	1.00	1.00	1.00	1.00
DEU	0.89	0.88	0.89	0.91	0.91	0.90	0.88	0.87	0.86	0.84	0.84
DNK	1.00	1.00	1.00	1.00	1.00	0.99	0.99	0.99	0.99	0.99	0.98
ESP	0.81	0.82	0.85	0.89	0.91	0.91	0.90	0.88	0.85	0.83	0.80
FIN	1.00	1.00	1.00	1.00	1.00	1.00	1.00	1.00	1.00	1.00	1.00
FRA	0.81	0.80	0.78	0.76	0.74	0.71	0.70	0.72	0.76	0.81	0.82
GBR	0.72	0.70	0.71	0.73	0.75	0.78	0.81	0.84	0.89	0.94	0.97
IRL	0.94	0.95	0.96	0.97	0.99	0.99	1.00	1.00	1.00	0.99	0.99
ITA	0.76	0.76	0.77	0.80	0.84	0.88	0.92	0.94	0.95	0.97	0.98
JPN	1.00	1.00	1.00	1.00	1.00	1.00	1.00	1.00	1.00	1.00	1.00
KOR	1.00	1.00	0.99	0.99	0.99	0.99	0.99	0.99	0.99	0.98	0.97
NLD	1.00	1.00	1.00	1.00	1.00	1.00	1.00	1.00	1.00	1.00	1.00
NOR	1.00	1.00	1.00	1.00	1.00	1.00	1.00	1.00	1.00	1.00	1.00
SVN	1.00	1.00	1.00	1.00	1.00	1.00	1.00	1.00	1.00	1.00	1.00
SWE	1.00	1.00	1.00	1.00	1.00	1.00	1.00	1.00	1.00	1.00	1.00

	1–12	2–12	3–12	4–12	5–12	6–12	7–12	8–12	9–12	10–12	11–12
*Government response stringency index*											
AUS	59.91	63.59	68.01	70.32	70.54	70.17	69.67	68.98	68.86	68.67	68.29
AUT	72.07	71.21	70.18	68.93	67.36	65.34	63.27	60.92	59.26	57.41	56.48
BEL	77.08	79.46	79.26	79.01	78.7	78.31	77.78	77.04	75.93	75	75
CAN	69.95	72.35	72.32	72.28	71.99	71.89	71.76	71.57	71.3	70.83	70.83
CZE	61.27	59.34	57.04	55.15	52.78	51.19	50.16	48.71	46.53	42.9	41.67
DEU	69.64	72.14	71.67	71.09	70.37	69.44	68.21	66.48	63.89	61.26	59.72
DNK	67.75	67.34	66.85	66.26	65.51	64.55	63.89	62.96	61.58	60.19	57.41
ESP	78.86	81.86	82.87	84.11	83.97	83.8	83.57	83.24	82.76	81.94	81.25
FIN	59.49	58.75	57.87	56.79	55.44	54.37	53.4	52.04	50	48.76	46.3
FRA	67.13	72.47	78.61	82.76	87.96	87.96	87.96	87.96	87.96	87.96	87.96
GBR	69.14	74.24	77.96	77.78	77.55	77.25	76.85	76.3	75.47	74.08	71.3
IRL	81.94	85.02	88.7	89.09	88.89	88.62	88.27	87.78	87.04	85.8	83.33
ITA	86.42	87.92	89.26	89.71	89.47	89.16	88.74	88.15	86.81	84.57	80.1
JPN	41.28	43.27	44.17	44.24	44.33	44.84	45.52	46.48	46.76	47.22	47.22
KOR	62.66	63.3	64.08	65.02	66.21	64.82	62.97	59.08	53.24	43.52	43.52
NLD	73.38	76.26	76.85	77.16	76.85	76.46	75.93	75.19	74.08	72.22	68.52
NOR	68.98	69.53	68.52	67.28	65.74	63.76	61.11	59.81	57.87	54.63	54.63
SVN	72.92	72.39	70.65	68.52	65.86	62.43	58.49	52.96	47.46	41.98	40.51
SWE	60.72	63.04	64.26	64.81	64.81	64.81	64.81	64.81	64.81	64.81	64.81
	1–12	2–12	3–12	4–12	5–12	6–12	7–12	8–12	9–12	10–12	11–12
	– 0.45	– 0.5	– 0.54	– 0.53	– 0.54	– 0.5	– 0.46	– 0.44	– 0.43	– 0.33	– 0.28

## Footnotes

1 Note: Short-time work is reported in the Assessment Capacities Project [6] database for Germany (line 699, 5392), Switzerland (line 4799, 9804), Spain (line 5162) and Sweden (line 6157).
